# PW03-030 – Collecting patients data to inform genetic studies

**DOI:** 10.1186/1546-0096-11-S1-A256

**Published:** 2013-11-08

**Authors:** AK Bybee, F Aslam, E Majdoub, D Rowczenio, A Baginkska, R Al-Nackkash, W Taylor, P Hawkins, H Lachmann

**Affiliations:** 1 National Amyloidosis Centre, UCL, UK; 2 Software Development, Zsah Ltd., London, UK

## Introduction

Our centre performed nearly 6000 genetic tests in 2011, in 11 genes, with a year on year increase of about 20% per year over 5 years. In half of the patients genetic testing was used as pre-screening before referral to clinic. A good patient history is essential for proper diagnosis and treatment of HRFS (hereditary recurrent fever syndromes), and genetic testing is a more useful adjunct when guided by relevant symptoms. In order to guide the testing strategy and provide more helpful interpretation of results, we asked the referring physicians to provide detailed information on the patient’s family history, symptoms, etc. The approach to collecting this information has evolved over several years, and we describe our experience.

## Objectives

To improve and streamline the collection of clinical data for correlation with genetic test results.

## Methods

Questions included description of family history, ethnic origin, attack parameters, precipitants and age at onset, 13 symptom areas (including AA amyloidosis), measures of acute phase response, and treatments, dose and effect s. Initially (Stage 1) this information was elicited using a paper proforma, designed to fit on a single side of A4 paper, and was filled in by the referring physician. These were scanned into our records and were accessed as needed, but the information contained could neither be searched nor filtered, making finding correlations and trends difficult. Later (Stage 2), the referring doctor could access a web link, provided by us on request, and could answer these questions online. The referring physician selected answers from drop-down boxes, with an option to write in some free text comments on the paper form to accompany the specimen. Further refinement was added in mid-2012 (Stage 3) with a web based approach, linked directly from the Centre website to allow the referring physician to request a test and answer the questions immediately. More detailed questions particularly about treatments were now included.

## Results

Referring physicians were very cooperative but in Stage 1 often obtained forms well after the patient consultation, meaning many useful details may have been omitted. Initial impressions are that the smoother operation of the web-based approach (Stage 2 and 3) is reflected in the increasing use of the online submission method, and seems particularly popular with non-UK referrals. The specimen reception staff took over the operation of the system with little need to consult with scarce IT staff, and the data collected is easier to access and is searchable. Negative feedback has been limited to a few problems mainly with access local to the referring physicians.

## Conclusion

Making the resulting data on the patient population searchable simplified correlation of the information provided with the genetic test findings, and has a immediate benefit of focussing scarce genetic testing resources to where they are most likely to be useful. In the future, technical advances may make genomic information much more plentiful; the concurrent development of good symptom curation using proper ontology will be an important tool for diagnosing and treating HRFS.

## Disclosure of interest

None declared

